# Impact of Aging on the Auditory System and Related Cognitive Functions: A Narrative Review

**DOI:** 10.3389/fnins.2018.00125

**Published:** 2018-03-05

**Authors:** Dona M. P. Jayakody, Peter L. Friedland, Ralph N. Martins, Hamid R. Sohrabi

**Affiliations:** ^1^Clinical Research, Ear Science Institute Australia, Subiaco, WA, Australia; ^2^School of Surgery, University of Western Australia, Perth, WA, Australia; ^3^School of Medicine, University of Notre Dame Australia, Fremantle, WA, Australia; ^4^Biomedical Sciences, Macquarie University, Sydney, NSW, Australia; ^5^School of Medical and Health Sciences, Edith Cowan University, Joondalup, WA, Australia

**Keywords:** age-related hearing loss, presbycusis, aging, auditory system, cognitive functions, dementia

## Abstract

Age-related hearing loss (ARHL), presbycusis, is a chronic health condition that affects approximately one-third of the world's population. The peripheral and central hearing alterations associated with age-related hearing loss have a profound impact on perception of verbal and non-verbal auditory stimuli. The high prevalence of hearing loss in the older adults corresponds to the increased frequency of dementia in this population. Therefore, researchers have focused their attention on age-related central effects that occur independent of the peripheral hearing loss as well as central effects of peripheral hearing loss and its association with cognitive decline and dementia. Here we review the current evidence for the age-related changes of the peripheral and central auditory system and the relationship between hearing loss and pathological cognitive decline and dementia. Furthermore, there is a paucity of evidence on the relationship between ARHL and established biomarkers of Alzheimer's disease, as the most common cause of dementia. Such studies are critical to be able to consider any causal relationship between dementia and ARHL. While this narrative review will examine the pathophysiological alterations in both the peripheral and central auditory system and its clinical implications, the question remains unanswered whether hearing loss causes cognitive impairment or vice versa.

## Introduction

Age-related hearing loss (ARHL) or Presbycusis, used interchangeably in this paper, is a common chronic health problem affecting individuals above 65 years old (WHO, 2012). ARHL is defined as a progressive, bilateral, and symmetrical hearing loss primarily observed in the high frequency region (Fetoni et al., 2011). Gates and Mills (2005) defined ARHL as a multifactorial disorder affecting hearing sensitivity varying from mild to substantial, resulting from lifetime insults to the auditory system. This review discusses the ARHL prevalence and risk factors, mechanisms of peripheral and central hearing loss, changes in the peripheral and central hearing pathway, and its consequences for speech understanding and cognition.

### Prevalence of ARHL

#### Frequencies

Most of the ARHL epidemiological studies have reported hearing loss prevalence data in the 500 Hz−4 kHz (Cruickshanks et al., 1998b; Gopinath et al., 2009), and/or between 4 and 8 kHz regions. However, age-related changes in hearing thresholds occur significantly earlier in the extended high frequencies, i.e., >8 kHz (Matthews et al., 1997; Sakamoto et al., 1998; Lee et al., 2005, 2012). Lee et al. (2012) reported two different aging process that impact the hearing thresholds across the entire frequency range: a slower process in the low frequencies (up to 4 kHz) and a faster process at higher frequencies (6–12.5 kHz) that in combination represent the thresholds spectrum across the entire hearing frequency range. For frequencies at 6, 8, 10, 11.2, and 12.5 kHz, the faster aging process starts at age 51, 47, 46, 36, and 30. In addition, for individuals aged 65–81 years old there was a decline of 0.7 dB per year at 0.25 kHz, increasing to 1.2 dB per year at 8 kHz, and 1.23 dB per year at 12 kHz (Lee et al., 2005). These results imply that by the time the first conventional pure tone audiogram (0.25–8 kHz) demonstrates ARHL, higher frequency loss (i.e., 10–12.5 kHz) could have already occurred. Therefore, preventive measures should be applied much earlier than previously thought, if the subsequent consequences of ARHL is to be minimized.

#### Race

National Health and Nutritional Examination Survey (NHANES), 2005-2006 data on participants aged > 70 years old revealed that on average, speech frequency (0.5–4 kHz), and high-frequency (3–8 kHz) PTA thresholds of the black participants were better than white participants by −5.8 (95% CI: −8.6 to −3.1), and −11.1 (95% CI: −13.9 to −8.2), respectively (Lin et al., 2011b). Agrawal et al. (2008) found that the odds of hearing loss was 70% lower in black vs. white individuals. Similarly, Helzner et al. (2005a) reported that from a sample of 2,052 participants, white participants were 63% more likely to have a hearing loss compared to black participants. The data from the National Health and Nutritional Examination Survey (NHANES) on 20–59 year old participants revealed that darker-skinned Hispanics showed better speech and high-frequency PTA compared to lighter-skinned Hispanics by an average of −2.5 dB hearing level (HL; 95% CI, −4.8 to −0.2) and −3.1 dB HL (95% CI, −5.3 to −0.8), respectively (Lin et al., 2012). These results indicate that melanocyte function may be protective against hearing loss in darker skin Hispanics (Lin et al., 2012).

#### Gender

For frequencies between 0.5–8 kHz, the prevalence of ARHL is two times higher in men compared to women (Agrawal et al., 2008; Gopinath et al., 2009). For example, bilateral hearing loss across the 60–69 years old age group is reported to be 43% and 20% in American men and women (Agrawal et al., 2008), and 29% and 17% in Australian men and women, (Gopinath et al., 2009), respectively. The onset of ARHL can be detected around the age of 30 years in men across the 0.5–8 kHz frequencies, however, the onset of ARHL in women occurs much later in life and varies across frequencies (Pearson et al., 1995). In addition, the rate of decline in hearing thresholds is much higher in men than women across 4–8 kHz frequencies, especially after the age of 50 years (Pearson et al., 1995). In contrary, hearing thresholds at 6 to 12 kHz frequencies decline at a significantly faster rate in women than men (Lee et al., 2005). Surprisingly, gender effect for extended high frequencies >8 kHz has not been reported for 10–65 year old individuals (Lee et al., 2012) or for 60–79 year age groups (Matthews et al., 1997).

### ARHL risk factors

Many attempts have been made to identify possible environmental and genetic risk factors associated with ARHL (Fetoni et al., 2011). Epidemiological studies have indicated that many of these factors are indeed very significant in the trajectory of ARHL. Yamasoba et al. (2013) identified four categories of risk factors associated with ARHL in humans: genetic predisposition, environment, health co-morbidities and cochlear aging. The first three risk factors associated with ARHL are reviewed here and cochlear aging will be discussed in the section on the impact of aging on the peripheral hearing system.

#### Genetics

Contribution of the genetic factors to ARHL in humans have been well-documented. Primary evidence comes from epidemiological studies on twin families in Swedish, North American, and Danish cohorts. A heritability of 0.47 for ARHL in Swedish mono-and dizygotic twins aged > 65 years (Karlsson et al., 1997), 0.4 for siblings (worse-ear average high-frequency thresholds) and 0.25 for parents and children (for worse ear middle and low frequency thresholds) have been reported (Framingham study; Gates et al., 1999). The heritability of hearing loss, as assessed using self-reports, for Danish twins aged 75 years old and over was at 0.4 (Christensen et al., 2001).

Genome wide association studies have reported a strong link between ARHL and single nucleotide polymorphisms (SNPs) located in GRM7, a gene encoding metabotropic glutamate receptor type 7 protein (mGluR7) (Friedman et al., 2009; Newman et al., 2012).

A number of genes and mutations responsible for monogenic non-syndromic hearing loss are linked to ARHL, including: (i) SNPs in 13-kb region in the middle of the KCNQ4, a gene which encodes a voltage-gated K-channel found in both outer and inner hair cells of cochlea (Van Eyken et al., 2006); (ii) 35delG mutation of GJB2, a gene that encodes gap junction proteins expressed in the inner ear (Van Eyken et al., 2007c); (iii) GRHL2, a gene that encodes a transcription factor expressed in cells lining cochlear duct (Lin et al., 2011); and (iv) mutation in MYO6, a gene that encodes myosin VI found in inner ear hair cells (Oonk et al., 2013).

In addition, genes that are linked to oxidative stress such as mutant allele (NAT2^*^6A), an isoform of N-acetyltransferases-encodes metabolism of reactive oxygen species (Ünal et al., 2005; Van Eyken et al., 2007b), and Glutathione S-transferases (GSTs)-encodes synthesis of glutathione antioxidant enzymes (Ateş et al., 2005) and mitochondrial dysfunction such as deletion of mitochondrial DNA 4,977 bp (Bai et al., 1997) and mitochondrial DNA haplogroups U and K are also reported to be associated with ARHL (Manwaring et al., 2007).

Furthermore, several candidate genes including apolipoprotein E (APOE) ε4 allele (O'Grady et al., 2007), variants of Endothelin-1 (EDN1) (Uchida et al., 2009) and polymorphisms of uncoupling proteins UCP2 Ala55Val (Sugiura et al., 2010) that are responsible for age-related diseases or clinical syndromes are found to be linked to ARHL (see also: Bovo et al., 2011).

In animal model studies, mice have been a useful animal model for studying the underlying cellular and genetic mechanism of human ARHL due to their small size, short life span, and genetic standardization (Ohlemiller, 2006). A number of loci that contribute to ARHL have been identified in inbred mice strains (Johnson et al., 2006). In various, inbred mouse strains an Ahl gene (age-related hearing loss) on chromosome 10 in C57BL/6J mice was found to be present (Johnson et al., 1997). Resistant alleles for Ahl 2, 4, and 8 were reported in the same mouse model (Johnson and Zheng, 2002; Johnson et al., 2008) and for Ahl 5 and 6 were found in CAST/Ei mice strains (Drayton and Noben-Trauth, 2006). Few other mouse models have been used to investigate the genetic mutations involved in ARHL (for examples see: Johnson et al., 2008; Sha et al., 2008; Ohlemiller, 2009). Further investigation into genetic bases of ARHL will further our understanding of pathogenesis processes that occur during aging and will also help provide more refined preclinical models of the disease.

#### Environmental factors

A variety of environmental risk factors such as exposure to industrial chemicals (Campo et al., 2013), occupational (Helzner et al., 2005a; Fransen et al., 2008), and recreational noise exposure (Clark, 1991) are reported to be associated with ARHL. Synergistic effects of simultaneous exposure to industrial chemicals and noise has a greater effect on ARHL than the impact of either one of the agents acting on its own (Morata et al., 2002; Sliwinska-Kowalska et al., 2004; Chang et al., 2006; Fuente and McPherson, 2006).

Exposure of mice to loud noises resulted acutely in substantial loss of cochlear afferent terminals and a slower degeneration of spiral ganglion cells, without significant cochlear hair cell loss or pure-tone hearing threshold loss (Kujawa and Liberman, 2009). Further, dysfunction of synaptic ribbons, which are essential for the accurate acoustic transmission between cochlear inner hair cells and post-synaptic afferent neurons (Fuchs et al., 2003) are documented in ARHL (Stamataki et al., 2006) and in noise-induced hearing loss (Kujawa and Liberman, 2009). For a review see Moser et al. (2013). Acoustic over-exposure also results in selective loss of low- and medium-spontaneous rate auditory nerve fibers in albino guinea pigs (Furman et al., 2013). The loss of spiral ganglion neurons (Kujawa and Liberman, 2006, 2009) and selective loss of low and medium spontaneous rate fibers (Furman et al., 2013) leads to poor temporal and frequency discrimination leading to impaired speech discrimination in noise in the absence of any audiometric loss (Plack et al., 2014). Such a hearing loss is known as “hidden hearing loss” (Plack et al., 2014). Kujawa and Liberman (2006) detected an increased vulnerability to primary neural degeneration of cochlea from mice that were exposed to noise at a younger age. Interestingly, genes (Ahl/Ahl) associated with ARHL increase the susceptibility to noise-induced hearing loss at a younger age in mice (Erway et al., 1996). These findings support the interaction between genetic predisposition to ARHL, noise and ARHL and should be taken into account in future preventive clinical trials.

#### Health co-morbidities

A number of health-related factors are reported to either increase the risk of ARHL or in fact provide protection against its development. Risk factors increasing ARHL include smoking (Helzner et al., 2005a; Nomura et al., 2005; Fransen et al., 2008) cardiovascular disease (Helzner et al., 2011), Type II Diabetes Mellitus (Frisina et al., 2006; Mitchell et al., 2009), and subclinical atherosclerosis (Fischer et al., 2015) are reported to increase the risk of ARHL. A number of oto-toxic medication are associated with irreversible hearing loss (Koegel, 1985; Brummett and Fox, 1989; Kwong et al., 1996). In contrast, moderate alcohol consumption (Popelka et al., 2000; Helzner et al., 2005a; Fransen et al., 2008), high bone mineral density (Helzner et al., 2005b), and dietary restrictions (Seidman et al., 2002) are described as to provide some protective impact against ARHL.

### The mechanisms of aging in the peripheral auditory system

The inner ear is more vulnerable to age-related changes than both the external and middle ear (Schmiedt, 2010). Hence, pathophysiological changes related to cochlea and afferent neurons are discussed here. Based on underlying case history information, audiometric configurations, and temporal bone analyses, Schuknecht (1974) divided ARHL into four distinct classes: sensory (loss of hair cells and supporting cells), neural (loss of afferent neurons), strial (atrophy of cochlear lateral wall and Stria vascularis), and cochlear conductive (cochlear atrophy mainly basilar membrane and organ of Corti). Rapid high-frequency sloping hearing loss, flat hearing loss, poor word discrimination and gradual hearing loss with no pathological evidence are characteristics of sensory, strial, neural, and cochlear conductive presbycusis, respectively (Schuknecht and Gacek, 1993). Later, Schuknecht and Gacek (1993) added two more categories: (i) mixed-consisting of a mixture of pathological characteristics and (ii) indeterminate-consisting of none of the aforementioned pathological characteristics. Contrary to the above-mentioned findings, Allen and Eddins (2010) reported that hearing phenotypes do not naturally form distinct classes of ARHL and rather form a continuum. However, subtypes of ARHL can still be identified if the distribution of data are in the extremes of either flat or sloping loss (Allen and Eddins, 2010).

Evidence for age-related pathological changes were reported in human temporal bone studies as well as in animal models. Histopathological studies have reported progressive loss and degeneration of spiral ganglion cells (Mills et al., 2006b; Hinojosa and Nelson, 2011), loss of nerve fibers in spiral lamina (Belal, 1975; Mills et al., 2006a) and hypertrophy of the internal elastic lamina of the internal auditory artery (Belal, 1975) in aged adults. Progressive outer hair cell loss with no degeneration of the cells have been reported in Stria vascularis (Sha et al., 2008), spiral ganglion cell degeneration (Keithley et al., 2004), and degeneration of organ of Corti and afferent neurons (Ohlemiller and Gagnon, 2004) in mouse models. Loss of outer hair cells and detachment of the stria vascularis from the spiral ligament was seen in aging Fisher rat F344 models (Buckiova et al., 2007). Loss of marginal and intermediate cells of Stria vascularis (Gates and Mills, 2005) and loss of Stria capillaries (Gratton and Schulte, 1995) were noted in aged gerbil models.

Two most prominent physiological changes have been observed in the aging cochlear: decline in cochlear endolymphatic potentials (EP) and increase in action potential (CAP) of the auditory nerve (Gates and Mills, 2005). Recent studies have concluded that metabolic/strial presbycusis or degenerative changes in the lateral wall and stria vascularis as the predominant cause of ARHL (Gates and Mills, 2005; Ohlemiller, 2009). The above mentioned pathological changes of the stria vascularis leads to loss of expression of key ion transport enzymes, such as Na+, K+-ATPase, and the Na+, K+, Cl− co-transporter, impairment in outer hair cell functions and decline in endolymphatic potential (EP) values (Salt et al., 1987; Wangemann, 2002). The EP provides voltage for the cochlear amplifier, thus, a decline in EP results in impairment in cochlear amplifier ultimately resulting in poor hearing thresholds (Schmiedt et al., 2002; Gates and Mills, 2005).

Another physiological impairment observed in aging cochlear is asynchronous firing of the auditory nerve fibers as indicated by increased thresholds of the compound action potential of the auditory nerve (Gates and Mills, 2005). A combination of pathological changes of the spiral ganglion cells and synaptic connections between the inner hair cells and afferents and decline in EP is thought to be one of the underlying causes of the auditory nerve malfunction (Gates and Mills, 2005).

Yamasoba et al. (2013) proposed a model to explain the development of ARHL. According to this model, a number factors (aging, genetic, environmental, health co-morbidity) and their interactions contribute to ARHL. Mitochondrial dysfunction associated with reactive oxygen species (ROS) plays a major role in the age-related cochlear cells degeneration (Cheng et al., 2005; Someya and Prolla, 2010). Noise trauma also generate excess of ROS in the cochlea (Henderson et al., 2006) triggering necrotic or apoptic cell death in cochlea (Henderson et al., 2006). Oxidative stress can be accelerated by hypoxic situations resulting from decline in cochlear blood supply due to noise trauma (Wen et al., 2017) or cardiovascular diseases (Nomiya et al., 2008) and genetic factors (Uchida et al., 2009), Ototoxic medication (Tabuchi et al., 2011), and health co-morbidity (Cruickshanks et al., 1998a) factors. Long-duration mitochondrial function leads to Bak-dependent apoptosis of the cochlear cells leading to increase in hearing thresholds (Someya and Prolla, 2010). Details of ARHL environmental and health-related co-morbid risk factors are described in Environmental Factors and Health Co-morbidities. Genetic investigations have identified a number genes associated with cochlear aging (see section Genetics). In addition, genes that are associated with antioxidant protection, mitochondrial dysfunction (see section Genetics), also contribute to the cochlear hair cell dysfunction.

### The mechanisms of aging in the central auditory system

**Figure 1:**
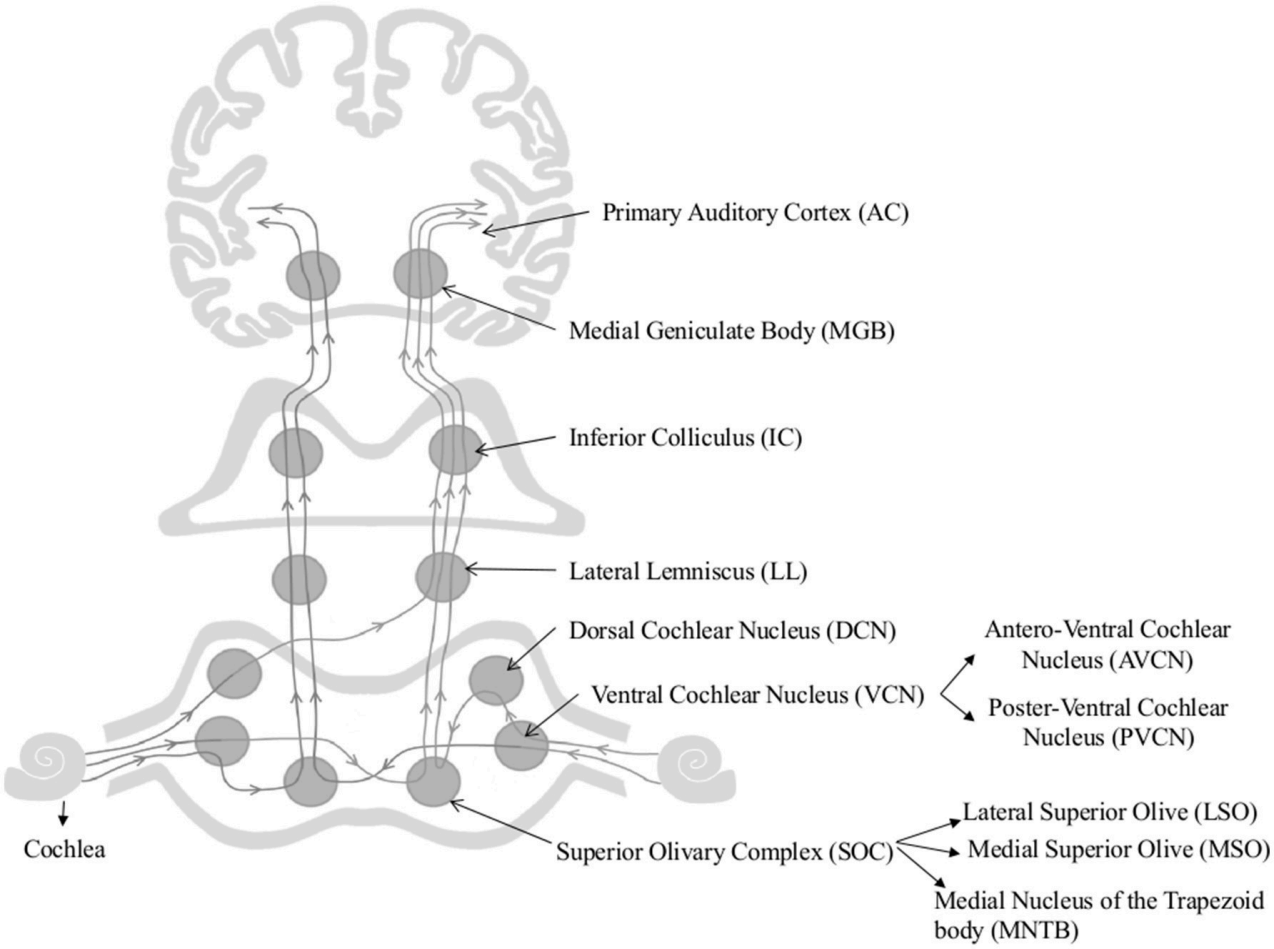
Schematic diagram of the auditory pathway. This schematic diagram represents ipsi and contralateral auditory pathways from cochlea to the auditory cortex. Main nuclei of the central auditory pathway, namely Superior Olivary Complex (SOC), Dorsal and Ventral Cochlear Nuclei (DCN & VCN), Lateral lemniscus (LL), Inferior Colliculus (IC), Medial Geniculate body (MGB), and Primary Auditory Cortex (AC) are shown in the figure.

The age-associated changes reported in cochlear nucleus and the auditory cortex have also been a focus of research. As Figure 1 shows afferent auditory fibers from the cochlea terminate at the cochlear nucleus (CN) complex which includes the anteroventral cochlear nucleus (AVCN), posteroventral cochlear nucleus (PVCN), and dorsal cochlear nucleus (DCN) (Brawer et al., 1974). First stage of centralized auditory processing occurs at the CN complex (Schmiedt, 2010). AVCN and PVCN carry out the spectral analysis of the auditory signal (Rhode and Greenberg, 1994) and AVCN and DCN process the localization cues (May, 2000). Both animal and human models have been used to investigate the underlying patho- physiological changes of the central auditory system associated with aging. The alterations in animal models from the CN to the auditory cortex are discussed first.

#### Evidence from animal studies

A number of important age-associated changes of the CN has been reported in animal models, briefly including: (i) decline in overall volume, total number, and neuron size of octopus cells in PVCN (C57 and CBA mice; Willott et al., 1992, 1994); (ii) decline in glycine mediated inhibition in DCN reported in Fisher-344 rats (Caspary, 2005); (iii) increase in total number and percentage of calcium binding protein (parvalbumin and calbindin) positive neurons in DCN and PVCN reported in C57BL/6J mice (Idrizbegovic et al., 2004); (iv) increase in the number of parvalbumin and nicotinamide adenine dinucleotide hydrogen phosphate diaphorase (NADPH-d) positive neurons in PVCN (Rhesus Macaque; Gray et al., 2014a); (v) decline in spontaneous miniature excitatory post-synaptic currents (mEPSC) in the high-frequency regions of AVCN [DBA mice] (Wang and Manis, 2005); and finally, (vi) age-related decline in processing of the temporal properties of the complex signals in DCN (e.g., Fischer Brown Norway rats; Schatteman et al., 2008). It is thought that age- associated changes in calcium binding proteins disrupt the calcium homeostasis and could lead to impaired synaptic transmission, reduced neural plasticity, and degeneration of neurons (Mattson, 2007). Spherical and globular bushy cells of the AVCN are involved in processing inter-aural time difference cues which are essential for sound localization in horizontal plane (Carney, 1990), hence, age-related alterations that occur in synaptic transmission in AVCN cells could result in poor sound localization and pitch detection of the acoustic signals. Furthermore, changes in processing in DCN could underlie the temporal deficits observed in older adults (Schatteman et al., 2008).

Only a handful of studies on the effect of aging of the superior olivary complex (SOC) are available. The SOC comprises three main nuclei, namely the medial and lateral superior olive (MSO and LSO, respectively) and medial nucleus of the trapezoid body (MNTB), (Harrison and Warr, 1962). The SOC plays a vital role in sound localization (Masterton, 1992) by processing inter-aural temporal and intensity differences (Grothe, 2000) and conveying the information to higher [lateral lemniscus (LL), inferior colliculi (IC), and medial geniculate nucleus (MGB)] and lower (to the cochlea and CN) centers of the auditory pathway (Oliver, 2000; Thompson and Schofield, 2000). Age-associated axonal and dendritic degeneration and loss of synaptic terminals have been observed in Sprague-Dawley rat medial nucleus of the trapezoid body (Casey and Feldman, 1982, 1985, 1988), however, no neuronal loss has been reported in LOS and MSO of aged Fischer 344 rats (Casey, 1990). Additionally, an age-related increase in parvalbumin and NADPH-d have been reported in MSO of rhesus macaques, but not in LSO and MNTB, suggesting an underlying compensatory mechanism to account for the poor auditory processing ability of the aging non-human primates (Gray et al., 2014b).

The inferior colliculus (IC) is considered one of the major convergence centers for both mono-and-binaural auditory information (Figure 1). It simultaneously processes the acoustic features of complex signals along with the other relay stations of the auditory pathway (Schmiedt, 2010). Age-associated cytochemical changes of the IC include: (i) a marked decline in GABA mediated inhibition (Burianova et al., 2009); (ii) increase in parvalbumin positive neurons (Engle et al., 2014); (iii) decline in the number of calbindin and calretinin positive neurons (Ouda and Syka, 2012); and (iv) decline in number of SMI-32-immunoreactive neurons and levels of non-phosphorylated neuro-filament proteins (Burianová et al., 2015). These changes influence the temporal processing of acoustical stimuli and reported to be contributing to central presbycusis (Ouda and Syka, 2012; Ouda et al., 2012).

Auditory thalamus/medial geniculate body (MGB) neurons transmit auditory stimuli to cortex and other subcortical structures by filtering and enhancing acoustic features of the auditory signal (Figure 1) (Bartlett, 2013). Gamma-aminobutyric acid (GABA) mediated inhibitory inputs are fundamental for auditory processing in the MGB (Cotillon-Williams et al., 2008). This inhibitory neurotransmission is obtained through synaptic and high-affinity GABAA receptors (Richardson et al., 2013). More than 45% reduction in GABAA receptor density and GABAA receptor mediated tonic whole cell Cl− currents was found in aged rat MGB. In addition, an increase in the number of parvalbumin positive neurons have also been reported in aged rhesus macaque MGB (Gray et al., 2013). Ouda et al. (2012) reported a decrease in calbindin positive neurons in Long Evans and -Fischer 344 rat MGB. These results are indicative of significant age-dependent deficits in the inhibitory neurotransmission within the MGB. Burianová et al. (2015) failed to observe age-associated decline in total number of neurons in IC, MGB, or primary auditory cortex (A1) of Long Evans and Fischer F344 rats. Temporal processing speed is affected by the senescent changes that occur in the cortex but not in the auditory thalamus or midbrain (Mendelson and Ricketts, 2001; Lee et al., 2002). Burianová et al. (2015) reported decline in number of neurons containing non-phosphorylated neuro-filaments and their protein levels, which are known for their conductance properties in the central auditory system. Overall, these changes may negatively impact processing of complex auditory signals in older adults (Richardson et al., 2013).

A number of animal experiments have reported age-related changes of A1. These include, (i) decline in the number of calcium binding positive neurons in rat A1 (Ouellet and de Villers-Sidani, 2014); (ii) decrease in the number and firing of V/U-shaped receptive field neurons in A1(Turner, 2005a); (iii) decline in number of neurons containing non- phosphorylated neuro-filaments and proteins (Burianová et al., 2015); and increase in (iv) spontaneous (29%), peak (24%), and steady state (38%) neuronal response rates in A1-layer V (Turner, 2005a); and (v) the number and firing of complex receptor field neurons in the A1-layer V (Turner, 2005a). These findings indicate that age-dependant changes in A1-layer V neurons could be responsible for diminished signal/noise coding (Turner, 2005a). Further, degradation of processing between cortical areas is reported to contribute to cognitive decline associated with the aging process (Juarez-Salinas et al., 2010). Cytochemical findings in C57BL/6J mice suggest that age-related alterations of glial cells in A1 are exacerbated by peripheral hearing loss (Tremblay and Burkhard, 2012). The A1 shows age-related decreases in pre- and post-synaptic GABA neurotransmission (Caspary et al., 2013). A significant age-associated decline in the number and density of GAD65 and 67 immuno-reactive neurons and GAD67 proteins in A1 layers have been observed in aged rats compared to their young adult controls (Ling et al., 2005; Burianova et al., 2009). These changes are accompanied by age-dependant alterations in the expression of GABAA receptor subunits. Together, these findings support the notion that age-related alterations of GABA neurotransmission alter the temporal coding properties of the primary auditory cortex. (Šuta et al., 2011) provided further evidence for age-related decline in temporal processing in Long Evans rats through behavioral and electrophysiological experimental measures. In sum, current animal model studies suggest that temporal processing of acoustical signals is associated with aging of the central auditory system and thus considered a major contributor to the poor speech perception skills seen in older adults (Ling et al., 2005; Caspary et al., 2013).

Finally, a number of animal experiments suggest that age-related pathophysiological changes of the central auditory system are further exacerbated by the peripheral hearing loss. For example, tonotopic reorganization of central auditory system following peripheral hearing loss has been reported in several animal experiments (Willott, 1984, 1986; Willott et al., 1993). Robertson and Irvine (1989) provided convincing evidence for cortical reorganization in guinea pigs 35–81 days following unilateral cochlear lesions. The auditory cortical areas that represented the frequencies of the damaged cochlea were responsive to the sound frequencies adjacent to the damaged frequency range. Response thresholds of the characteristic frequencies of these reorganized cortical neurones were close to their normal thresholds (Mean difference = −3.8 dB). Similar findings have been observed few hours after a cochlear lesion except that the reorganized cortical neurons had higher response thresholds compared to their normal thresholds (mean difference 31.7 dB).

#### Evidence from human studies

Recent human studies have utilized several different neuroimaging techniques to investigate the age-related changes of the human central nervous system including magnetic resonance imaging (MRI) to measure brain volumes and MRI spectroscopy for delineating neurochemical and metabolic changes (Ouda et al., 2015). Some of studies on the brain-related changes associated with auditory system will be summarized in this section.

##### Central auditory pathway changes due to healthy aging process

Structural MRI studies have shown a reduction in both gray (Raz et al., 1997, 2004; Lemaitre et al., 2012) and white matter volumes (Silver et al., 1997; Raz et al., 2004, 2007) and cortical thinning due to aging (Lemaitre et al., 2012). Hedman et al. (2012) reviewed 56 longitudinal MRI studies to investigate the age-related brain volume changes in healthy adults. Results revealed annual brain volume loss of 0.2% at 35 years old reaching 0.5% by the age of 60 years. Greater than 0.5% annual brain volume loss observed after 60 years. Age-associated marked decline in temporal lobe volume (Scahill et al., 2003), hippocampal volume (Scahill et al., 2003; Raz et al., 2004), and prefrontal cortex (Raz et al., 1997) have been reported in region-specific imaging studies. Alterations in the above mentioned neural networks are implicated in impaired cognitive functions such as verbal recognition memory (Fletcher and Henson, 2001; Henson, 2005), episodic visuospatial memory, learning and association ability (Sweeney et al., 2000), working memory and executive functions (Owen et al., 1995, 1996), and attention switching (Makhani et al., 2015).

MRI spectroscopy has been used to describe age-related neurochemical and metabolic changes of the central nervous system (Gujar et al., 2005; Gruber et al., 2008; Kirov et al., 2008; Reyngoudt et al., 2012; Gao et al., 2013, 2015). Age-related decrease in glutamate and N-acetyl aspartate levels have been observed in elderly participants with mild and expressed ARHL (Profant et al., 2013). An increase in lactate levels has been observed in elderly participants with expressed ARHL, however, no significant changes in GABA concentrations was noted between young and elderly participants(Profant et al., 2013). Contrary to these observations, Gao et al. (2015) reported significantly lower GABA+ concentrations in participants with ARHL as compared to normal hearing controls. Data from participants with ARHL revealed a significantly negative correlation between pure-tone audiometric average across 500–4,000 Hz and GABA+ concentrations (Gao et al., 2015). These results are consistent with the hypothesis of dysfunctional GABAergic neurotransmission in central auditory system in those with ARHL.

##### Changes in the central auditory pathway due to ARHL

Fewer studies have investigated the impact of ARHL on central auditory system using neuroimaging techniques. In a cohort of 126 participants aged between 56 and 86 years old with normal to severe sensorineural hearing loss, ARHL was shown to be independently associated with higher rate of decline in the entire brain volume and right temporal lobe volume (Lin et al., 2014). A summary of MRI studies revealed that the ARHL results in the changes of the central auditory pathway, including (i) decline in gray matter volume observed in superior and middle temporal gyri (Husain et al., 2011), superior and medial frontal gyrus (Husain et al., 2011; Boyen et al., 2013), primary auditory cortex (Peelle et al., 2011; Eckert et al., 2012), occipital lobe (Boyen et al., 2013), and hypothalamus (Boyen et al., 2013); (ii) a decline in both gray and white matter areas near auditory cortex (Husain et al., 2011); (iii) a decrease in fraction anisotropy values in SOC (Chang et al., 2004), LL & IC (Chang et al., 2004; Lin et al., 2008), and auditory cortex (Chang et al., 2004); and (iv) a decrease in fraction anisotropy values in a number of white matter tracks including right superior and inferior longitudinal fasciculi and corticospinal tract (Husain et al., 2011). Collectively, these findings suggest that ARHL not only results in secondary pathophysiological changes in the central auditory pathway, but also in the areas of the brain that are not directly involved in processing auditory stimuli. The underlying mechanism for the association of peripheral hearing loss and cortical tonotopic reorganization secondary to peripheral hearing loss may further compromise listening and comprehension abilities of older adults (Peelle et al., 2010, 2011).

## Consequences of ARHL

### Speech understanding

Difficulties expressed by older adults in understanding speech could be due to age-related deficits in peripheral and/or central auditory pathways (Gates and Mills, 2005). Among the peripheral factors that impact speech understanding, poor audibility resulting from cochlear pathology is considered to play a significant role (George et al., 2007). However, part of the problem in recognizing speech in older adults seem to be associated with temporal deficits resulting from aging and ARHL (Gordon-Salant and Fitzgibbons, 1993). Several studies have investigated the impact of aging on central auditory processing of speech stimuli (See Humes et al., 2010 for a review). These studies have used competing speech (Jerger et al., 1991; Gates et al., 2008), temporally-distorted speech (Gordon-Salant and Fitzgibbons, 2001), and/or binaural speech stimuli (Jerger et al., 1991; Gates et al., 2008). A review by Humes and Dubno (2010) concluded that aging significantly impairs all three speech test measures and hearing loss was the major factor on negatively impacting participants' performance on tasks that utilized competing and time compressed speech stimuli. The above mentioned temporal processing deficits could explain some of the difficulties faced by older adults, with or without hearing loss, in perceiving speech in challenging situations-i.e., accented speech (Gordon-Salant et al., 2013) and speech in noise or reverberation (Dubno et al., 1984; Gordon-Salant and Fitzgibbons, 1993). Further, speech comprehension in quiet and in noise also relies on cognitive functions (Pichora-Fuller et al., 1995). These include searched based attention (Pichora-Fuller and Singh, 2006), selective attention (Alain and Arnott, 2000), divided attention (Kemper et al., 2003), working memory (Pichora-Fuller et al., 1995), and processing speed (Pichora-Fuller and Souza, 2003).

### Dementia and cognitive impairment

There are two key components of the auditory system involved in the processing of incoming auditory stimuli; the peripheral hearing system and the central hearing system (Katz, 2015). Approximately 83% of adults aged 70 years and above suffer from a peripheral hearing loss (Cruickshanks et al., 1998b). Peripheral hearing loss not only affects the auditory processing of speech sounds but also the higher-level cognitive functions required to process linguistically demanding stimuli (Stewart and Wingfield, 2009; Peelle et al., 2011). For example, the hearing impaired individuals show difficulty in attending to working memory (Wingfield et al., 2006) and auditory and visual free recall tasks (Wingfield et al., 2006) and require longer latencies to make accurate perceptual judgments (Tun et al., 2010). Evidence from both cross-sectional (Valentijn et al., 2005; Tay et al., 2006; Jayakody et al., 2018a), and longitudinal (Valentijn et al., 2005; Lin et al., 2011a; Deal et al., 2016) studies found an association between peripheral hearing loss and cognitive impairment in older adults. A meta-analysis study reported an association between hearing loss and cognitive impairment, with the degree of cognitive impairment being significantly associated with the severity of both untreated and treated peripheral hearing loss (Taljaard et al., 2015). Further, an association between ARHL and risk of incident dementia (Lin et al., 2011a; Gallacher et al., 2012; Gurgel et al., 2014; Deal et al., 2016) and risk of incident Alzheimer's disease (AD) (Lin et al., 2011a) has also been reported. Most of the studies reported above have considered verbally loaded cognitive measures to assess the cognitive functions of older adults. Cumulative evidence strongly suggest that hearing loss could result in overestimation of the level of cognitive impairment in hearing impaired participants (Jorgensen, 2012; Dupuis et al., 2015). Therefore, non-verbal cognitive measures could be more appropriate for older adults with potential ARHL. A small number of recent studies that investigated the association between ARHL and cognitive impairment by removing auditory items of the cognitive screening tests (Dupuis et al., 2015) or using non-auditory tests of cognition (Lin et al., 2013, 2017; Wong et al., 2014; Jayakody et al., 2018a) have also reported an association between peripheral hearing loss and cognitive impairment.

In some individuals, difficulties experienced during comprehending speech in noisy backgrounds or competing speakers could be related to central auditory processing (CAP) difficulties rather than peripheral hearing deficits (Panza et al., 2015). Results from a number of longitudinal studies suggest that CAP in the absence of severe peripheral hearing loss is associated with high incidence of cognitive decline and dementia due to AD (Gates et al., 1996, 2002, 2008, 2011; Profant et al., 2015). These studies have demonstrated that individuals with central auditory dysfunction were at a significantly increased risk for incident dementia with hazard ratios ranging from 9.9 (95% confidence interval [CI], 3.6–26.7) to 23.3 (95% CI, 6.6–82.7) (Gates et al., 1996, 2011; Profant et al., 2015). Gates et al. (1996) reported that the relative risk of developing cognitive decline or dementia was twice higher for those with bilateral poor CAP scores as measured by Synthetic Identification-Ipsilateral Competing Message (SSI-ICM) scores compared to those with unilateral poor SSI-ICM scores. Furthermore, impaired central auditory functions have been observed five to ten years prior to an official AD diagnoses (Iliadou et al., 2003; Bateman et al., 2012).

Numerous attempts have been made to provide plausible explanations for the association between ARHL and cognitive decline. Cognitive load on perception hypothesis argues that the decline in cognitive capacity increases the cognitive load resulting in a sensory loss (CHABA, 1988; Lindenberger and Baltes, 1994). Sensory Deprivation hypothesis proposes that hearing loss leads to permanent deterioration in cognitive functions (CHABA, 1988; Lindenberger and Baltes, 1994; Schneider and Pichora-Fuller, 2000; Pichora-fuller, 2003; Lin et al., 2011b, 2013; Humes et al., 2013). Cortical reorganization following ARHL provides substantial evidence to support the sensory deprivation hypothesis (Husain et al., 2011; Peelle et al., 2011; Eckert et al., 2012; Boyen et al., 2013). Findings demonstrating that severe to profound hearing impaired children and young adults with a long duration of hearing impairment performed similar to their hearing peers on tests of intelligence seem to contradict the sensory deprivation hypothesis (Vernon, 2005). Hence, one could argue that hearing is not the sole contributor to cognitive impairment, rather one of the factors. Information degradation hypothesis postulates that hearing loss results in reversible cognitive decline (CHABA, 1988; Schneider and Pichora-Fuller, 2000; Pichora-fuller, 2003). Supportive evidence for the information degradation hypothesis can be found in a large number of existing research studies. In sum, these studies suggest that older adults rely more on cognitive resources to interpret degraded speech signals, resulting from hearing loss thereby placing more demand on executive functions and working memory (Pichora-Fuller et al., 1995; Pichora-fuller, 2003; Pichora-Fuller and Singh, 2006; Amichetti et al., 2013).

Common cause hypothesis argues that a common mechanism underlies age-associated changes observed in cognition, hearing, and other sensory modalities (CHABA, 1988; Lindenberger and Baltes, 1994; Baltes and Lindenberger, 1997). It has been suggested that general age-related neuropathological changes in the brain may actually explain this relationship, but current data do not support this general domain and function specific underlying mechanism theory (Lindenberger and Ghisletta, 2009). For example, when the relationship between hearing loss and memory was examined, a significant relationship was observed even after controlling for the effects of age and the type of task (e.g., verbal vs. visual), confirming hearing loss is an independent factor involved in cognitive decline (Lindenberger and Ghisletta, 2009).

Wayne and Johnsrude (2015) proposed a new framework with a common cause phenomena approach, where age-related changes of the brain result in decline of sensory (e.g., ARHL) and cognitive abilities. According to this hypothesis, the changes in sensory abilities increase the demand on the already strained cognitive system resulting in functional cognitive impairment (Wayne and Johnsrude, 2015).

Recently, a cognitive reserve hypothesis has been proposed to explain how individuals with similar neuropathological conditions differ substantially in their ability to make efficient use of brain reserve during tasks (Stern, 2002). Intelligence (Alexander et al., 1997) and higher education (Amieva et al., 2014), occupational level (Staff et al., 2004), participation in leisure activities (Scarmeas et al., 2001), and social networking (Fratiglioni et al., 2000) are considered to be contributing factors to the cognitive reserve. If sensory or cognitive demands exceeds the cognitive reserve capacity, it could lead to impairment in cognitive abilities (Wayne and Johnsrude, 2015). However, according to cognitive reserve hypothesis, the observed cognitive impairment could be reversed if the perceptual challenges were reduced (i.e., improved speech perception through amplification; Wayne and Johnsrude, 2015). In summary, no single hypothesis has been able to provide a comprehensive inference with regards to the causal relationship between ARHL and cognitive impairment.

Even though, ARHL is still not considered as a major risk factor for AD, it is a modifiable age-associated condition linked to dementia and late-life cognitive decline (Livingston et al., 2017). The Lancet International Commission on Dementia Prevention, Intervention, and Care has estimated that a third of AD diagnoses worldwide could be delayed or prevented, with early intervention and changes to lifestyle and utilizing available public health strategies (Livingston et al., 2017) such as post-lingual hearing loss correction. Furthermore, studies demonstrate that if the prevalence of each risk factor is reduced by only 10–20% per decade, the number of AD diagnoses could reduce by between 8.8 and 16.2 million worldwide by 2050 (Norton et al., 2014). As an example of potential changes in outcome measures following hearing loss correction, we have recently reported that cochlear implant recipients performed significantly better on cognitive function measures compared to similar individuals on a waiting list to receive cochlear implants (Jayakody et al., 2017). Others have also reported similar positive results following hearing loss improvement using hearing aids and cochlear implantation (Castiglione et al., 2016; Sonnet et al., 2017). Whether the correction of ARHL could significantly delay or arrest the late life pathological cognitive decline, dementia, or AD neurodegeneration is yet to be investigated. However, treating ARHL is extremely low risk, has significant health, social, and safety benefits beyond cognition, and there are enough evidence to discuss this option with ARHL patients (Golub, 2017).

### Frailty

ARHL is an important marker in frailty (Panza et al., 2015). Frailty has been defined as a clinical syndrome that contains three or more of the following symptoms: unintentional weight loss (10 lbs in past year), self-reported exhaustion, weakness (grip strength), slow walking speed, and low physical activity (Fried et al., 2001). Frailty increases the risk of detrimental health hazards such as falls (Speechley and Tinetti, 1991), institutionalization (Rockwood et al., 1996), hospitalization (Purser et al., 2006), and death (Ensrud et al., 2007). Interestingly, the risk of frailty in older adults with hearing loss increases by 63% and ARHL appears to be an independent risk factor for frailty with great risk of falls (Kamil et al., 2016). Further, ARHL has been shown to be associated with health-related outcomes linked to frailty, such as low level of physical activity (Gispen et al., 2014), slow gait speed (Li et al., 2013), and incident falls (Lin and Ferrucci, 2012). In addition, frailty is reported to be associated with cognitive impairment (Ávila-Funes et al., 2009; Yassuda et al., 2012), cognitive decline (Samper-Ternent et al., 2008; Auyeung et al., 2011) and incident dementia (Solfrizzi et al., 2013). See Robertson et al. (2013) for a review.

### Mental health and ARHL

Social isolation and loneliness is considered one of the risk factors for ARHL. Mick and Lin (2013) found a significant association between ARHL and social isolation in the 60–69 year old age group [odds ratio = 2.4, 95% CI = (1.36, 4.23) *p* = 0.003]. The association was more pronounced in females than in males (Ramage-Morin, 2016). ARHL is significantly associated with late-life depression, anxiety, and stress (Jayakody et al., 2018b). Depression is considered an early manifestation of dementia and AD (Panza et al., 2010). Accumulated evidence suggest that depression accelerates the aging process by increasing the incident risk factors of age-associated diseases such as cardiovascular diseases (Frasure-Smith et al., 1993), metabolic disturbances (McIntyre et al., 2007), and cognitive impairment (Lee et al., 2011; Dawes et al., 2015). Further, a bidirectional association between depression and late-life frailty has also been reported (Mezuk et al., 2012). Freret et al. (2015) proposed that an imbalance between brain reserve, resilience, neuroplasticity, and impaired pathophysiological mechanisms associated with aging, stress, and synaptic plasticity may lead to late-life depression. Although the pathophysiological mechanisms underlying depression, ARHL, and dementia remain to be further elucidated, a putative mechanism underlying both depression and dementia linked to neuro-inflammation and perfusion deficits in older adults (Maes et al., 2009; see Popa-Wagner et al., 2014, for a review).

**Figure 2:**
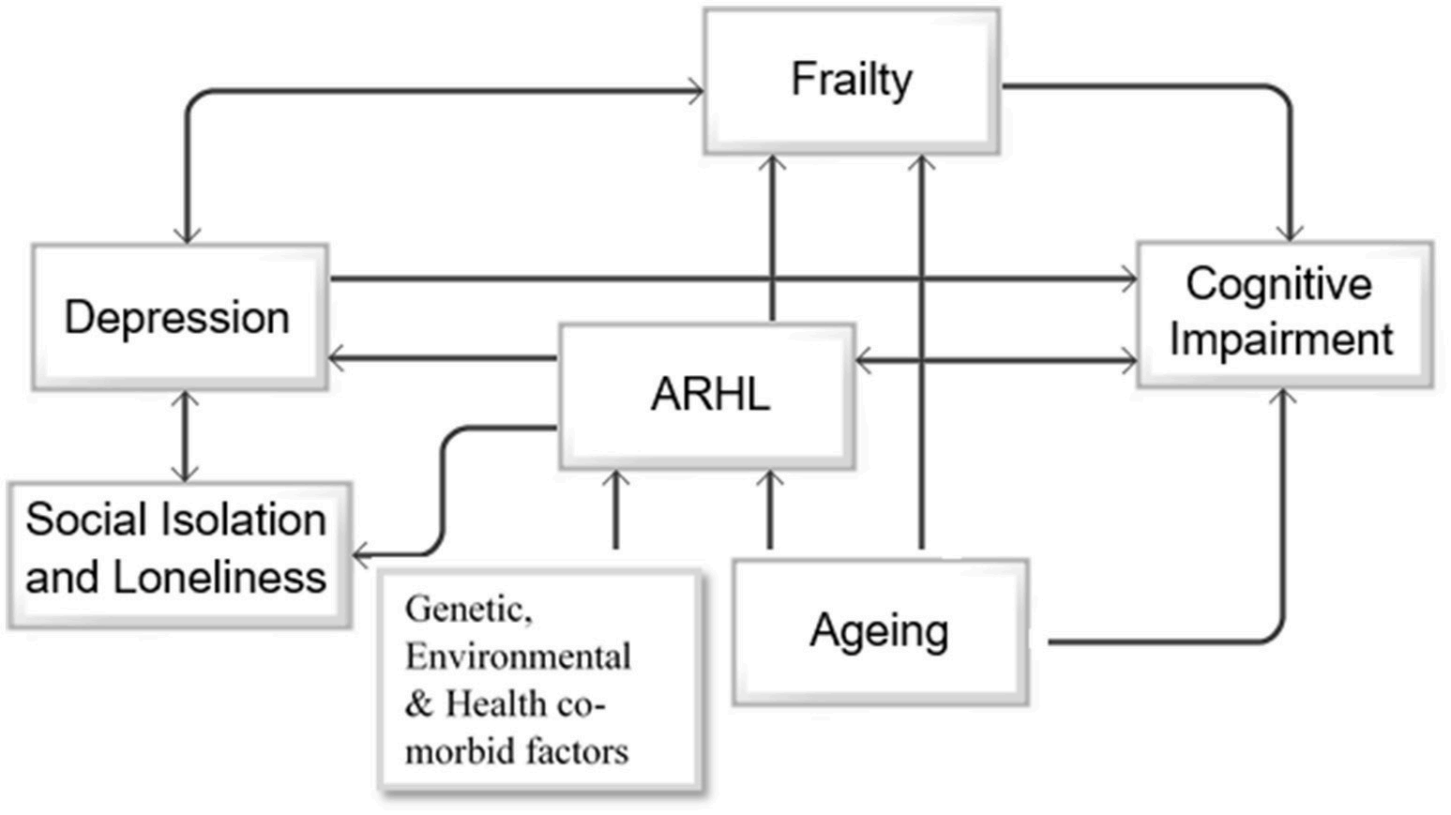
Directional associations between aging, ARHL, depression, social isolation and loneliness, frailty and cognitive impairment. The schematic diagram represents the unidirectional association between aging, age-related hearing loss (ARHL), and cognitive impairment, and unidirectional association between ARHL and social isolation, depression and frailty, and bidirectional association between depression and social isolation and depression and frailty.

Based on the above reported findings, there seems to be a bidirectional association between ARHL and cognitive impairment, depression and social isolation, depression, and frailty (Figure 2). As shown in the schematic approach in Figure 2, primary factors including aging, genetic predispositions, environmental factors, and medical and health related conditions accompany cognitive decline, ARHL, and frailty. Furthermore, ARHL increases the risk of depression and frailty, both of which are associated with age-related cognitive decline and future risk of dementia. As ARHL is the primary modifiable risk factor for cognitive decline, depression, and social isolation in this model, its treatment may, to some degree, improve the associated outcomes and change the trajectory of cognitive impairment. The diagram shown in Figure 2 is a simplified account of the relationship between various known factors with the understanding that so many are yet to be determined. However, this model provides a basic rationale to argue that treating ARHL will provide multiple benefits, each with significant implications for future trajectory of cognitive functioning in older adults.

## Shortcomings of the current literature and future direction

A number of limitations can be identified in the existing body of literature on neuro- patho-physiological changes in the peripheral and central auditory systems due to aging. As mentioned in the previous sections, ARHL is influenced by different factors including aging, lifestyle choices, genetics, and environmental factors (Van Eyken et al., 2007a), hence, pathophysiological consequences of ARHL cannot be studied in isolation. Most of the inferences on the aging human auditory system have been drawn from the results of animal experiments or post-mortem human temporal bone studies. Inadequate sample size across lifespan studies, lack of robust experimental designs, and a paucity of comprehensive case history information cannot be underestimated in the available human studies. Another limitation relates to the hearing assessments methods used in previously published ARHL studies. The majority have utilized selective audiometric data to identify the decline in hearing sensitivity. Even though it has been established that the ARHL primarily affects high-frequency hearing thresholds, most of the studies have reported either 3 PTA (i.e., average of pure-tone thresholds at 500 Hz, 1 and 2 kHz) or 4PTA (i.e., average of pure-tone thresholds at 500 Hz, 1, 2, and 4 kHz) data, thereby omitting the vital high-frequency information. Whilst it has been well-documented that older adults have difficulty with perceiving complex sounds (speech or music-in-noise; Kim et al., 2006; Alain et al., 2014), only a handful of studies have incorporated speech-perception-in-noise tests in their designs. Further, no study, to our knowledge, has used vocal or instrumental sounds in the presence of background accompaniment, thus failing to provide an accurate picture of the real-life difficulties faced by elderly participants with ARHL. Finally, hearing thresholds should be taken into consideration while assessing cognitive functions of older adults. Failure to do so could underestimate the level of cognitive functioning in older adults with a hearing (Jayakody et al., 2018a) and/or visual impairment (Dupuis et al., 2015).

In conclusion, aging results in pathological and physiological changes in both peripheral and central auditory systems. A number of genetic, environmental, and health co-morbid factors increase the risk of ARHL. Peripheral hearing loss further exacerbates the changes in the central auditory pathway. In this review, we have described the evidence for the association between ARHL and cognitive impairment, AD, depression, social isolation, and frailty. There is no substaintial evidence of how ARHL and AD-related biomarkers are associated prior to the clinical manifestation of the dementia syndrome. This is a necessary yet expensive research question that will inform our understanding of the statistical associations reported between the two. However, it is clear that ARHL represents a modifiable condition and a useful target for secondary prevention of cognitive impairment, frailty, social isolation, and depression in older adults. Further research is required to comprehensively address underlying mechanisms and determine whether hearing loss treatment could delay or arrest late life cognitive decline or dementia. Specifically, longitudinal studies including randomized clinical trials with representative samples of hearing aid, and hearing implant users and rehabilitation programs could further elucidate the association between ARHL and other components of our proposed model in Figure 2.

## Author contributions

All authors listed have made a substantial, direct and intellectual contribution to the work, and approved it for publication.

### Conflict of interest statement

DJ and PF have no conflicts of interest to declare. RM is the founder and Chief Scientific Officer of the Alzhyme and KaRa Minds Institute. HS has, and continues to receive remuneration from activities with Pfizer and Takeda Pharmaceuticals. Although grants and funds for our research were provided by public and private organizations, they in no way influenced any part of this review, its conclusions or the decision to submit this manuscript for publication to this journal.
